# Frightening" Resistant Clostridial Myonecrosis: A Case Report

**DOI:** 10.7759/cureus.4539

**Published:** 2019-04-24

**Authors:** Syed Adeel Hassan, Ali Akhtar, Maham Khan, Fahad N Sheikh, Hannan Asghar

**Affiliations:** 1 Internal Medicine, Dow University of Health Sciences, Karachi, PAK; 2 Internal Medicine, Pakistan Air Force Hospital, Islamabad, PAK; 3 Radiology, Armed Forces Institute of Radiology and Imaging, Islamabad, PAK; 4 Medicine, Sahiwal Medical College, Sahiwal, PAK; 5 Internal Medicine, Sahiwal Medical College, Sahiwal, PAK

**Keywords:** gas gangrene, myonecrosis, clostridium perfringens, hyperbaric oxygen, traumatic infection, anaerobic infection, alpha toxin, clostridium bifermentans, clostridium sordelli, crepitations

## Abstract

Clostridial myonecrosis is a diffuse necrotizing infection of deep soft tissues. It is known for its acute, rapid progression, poor prognosis, and high mortality. We report a case of traumatic clostridial myonecrosis, a 17-year-old, previously healthy female reported to our department with the complaint of pain in her left arm a week after she suffered a fall from her chair on to her left arm. Due to the injury sustained in the incident, under suspicion of a forearm fracture, a cast was applied to her left arm at a local polyclinic. However, after a few days, she reported unbearable pain, which led to the removal of the cast, and that is when diffuse crepitations due to gaseous accumulation were noted in her entire left upper limb. A week later, X-ray studies failed to reveal any fracture or abnormality. The patient was started on broadspectrum antibiotic coverage with intravenous (IV) benzylpenicillin, rifampicin, and clindamycin for a considerable period of time, but there was no improvement in her condition and the infection continued to spread into adjacent soft tissues, requiring intervention with hyperbaric oxygen therapy.

## Introduction

Clostridial myonecrosis, also known as true gas gangrene, is historically known to cause painfully challenging and debilitating infections in battlefield wounds. Initially, the disease was known as “malignant edema” up until 1882 by Pasteur, Koch, and Geffky but later was termed as gas gangrene by Moliere and Ponget. As reported, the incidence of infection in telluric battlefield wounds has noticed a drastic fall from 5% in World War 1 to a meager 0.1% in the post-Vietnam war era [1-2]. The gradual reduction in the incidence of cases can be owed to the rapid availability of appropriate antibiotics, improved antiseptic protocols, and wound management. In well-developed countries, such as the United States, the annual incidence of myonecrosis is about 1000 cases per year [1]. For purposes of clinical discussion, clostridial myonecrosis is subdivided into two types: traumatic and spontaneous [2]. The two most common organisms associated are Clostridium (C.) perfringens and Clostridium septicum, respectively. The spontaneous form of the disease tends to occur in individuals with immunocompromised states, such as diabetes, leukemia, neutropenia, and various forms of malignancy, and gastrointestinal abnormalities such as colorectal carcinoma [1]. Of the two, C. septicum is not a member of the normal human gut normal flora. Such disease states help promote an environment of pathological adaptation and dysbiosis, thereby favoring the survival and colonization of C. septicum in the gut [2]. We report a case of traumatic clostridial myonecrosis due to C. perfringens.

## Case presentation

**Figure 1:**
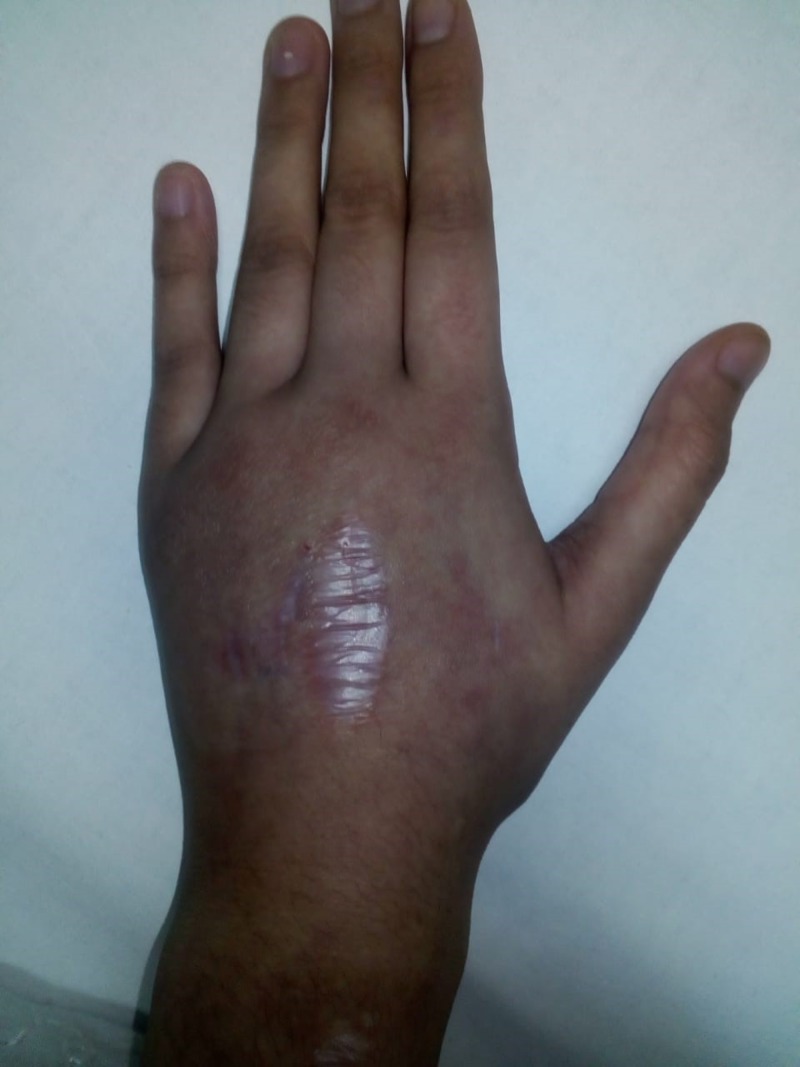
Left hand, post removal of the cast Note the early puckering of the skin associated with minimal swelling and gas accumulation.

**Figure 2:**
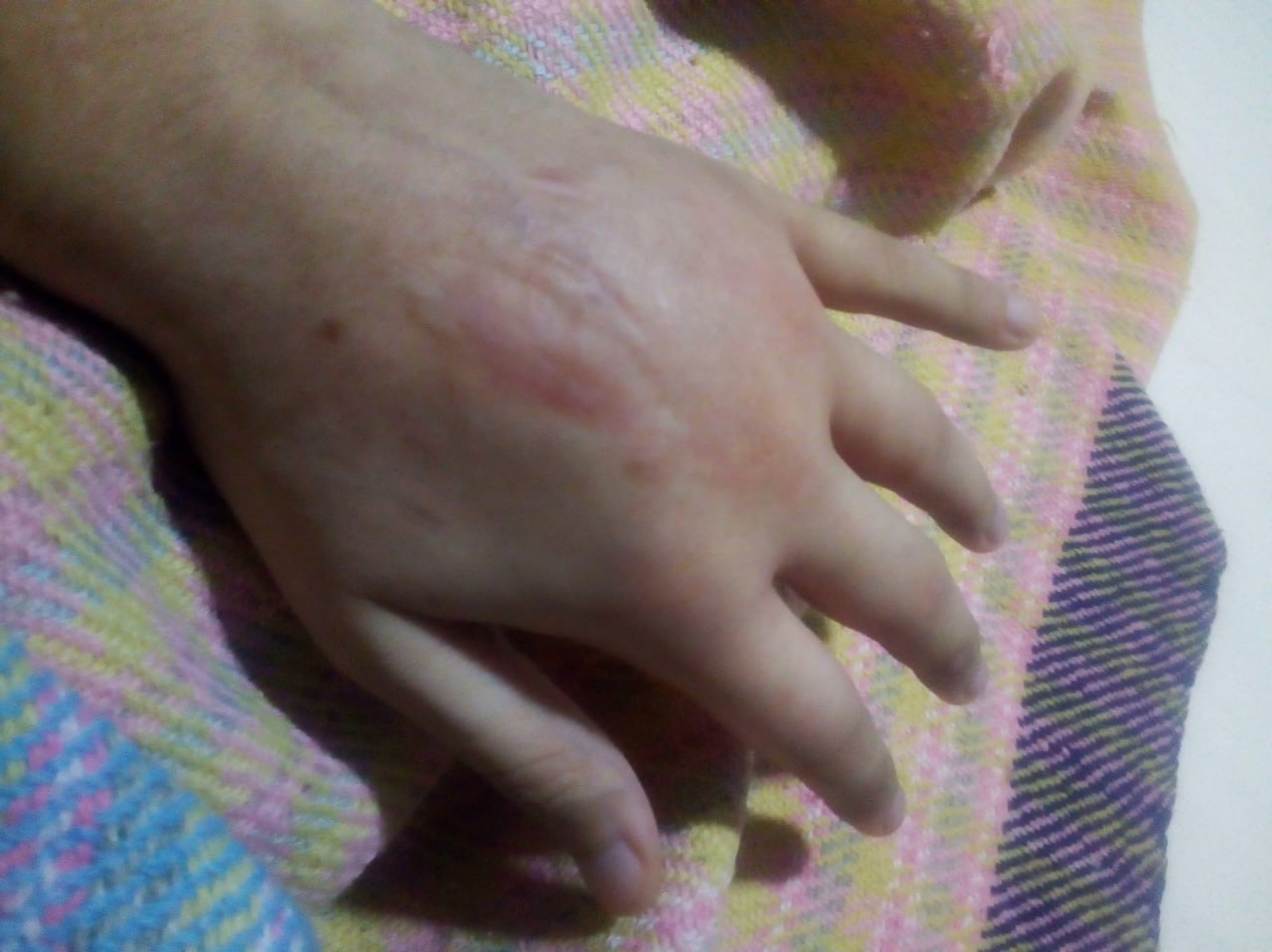
Left hand, few days after the removal of the cast Note the diffuse and rapid spread of tissue involvement. Also of significance is the marbling of the skin in the involved area due to gaseous accumulation.

Our patient is a 17-year-old, previously healthy female, who was admitted to our general medicine ward nine months ago with a history of a fall from her school chair. The patient suffered a minor scratch and experienced sudden sharp pain in her left hand but could not localize the pain site. Post the incident, she was taken to a local dispensary where a cast was applied to her left forearm due to suspicion of a fracture. However, despite the cast, her pain increased sharply in one week, which became intolerable. The X-ray done at the local dispensary did not reveal any fracture or abnormality in her hand. Upon removal of the cast, diffuse swelling was noted on the dorsal surface of her left hand with crepitations due to gas accumulation under her skin tissue, which was spreading upwards at a rapid pace (Figures 1-2).

She was immediately referred to a tertiary care hospital, where a skin biopsy confirmed gas gangrene by the culprit Clostridium perfringens. As per protocol, she was administered IV benzylpenicillin and was further instructed to comply with oral clindamycin and rifampicin for 22 days. However, no significant improvement was seen after an extensive 22-day regime. In fact, her condition had further deteriorated due to the accumulated gas under her skin, which now also involved other areas of her body such as the face, right hand, trunk, left leg, suprascapular region, and the pre and post-auricular as well as the thoracolumbar region. An early decision regarding amputation was made by the surgeon, but her parents had refused the surgery. Five escharotomies were performed along with the recommended course of extended spectrum antibiotics, but no significant improvement was seen and the patient continued to experience pain and gaseous crepitation at major areas of her body. Fortunately, hyperbaric oxygen therapy, along with antibiotics, was administered, which led to a significant improvement in her condition. However, after 40 sessions of hyperbaric oxygen, she started experiencing adverse effects such as ear blockage, bleeding from the root ends of her hair, and pain in the neck region and both upper limbs for a brief period of time; these were managed conservatively. Currently, our patient has undergone a total of 139 sessions of hyperbaric oxygen therapy along with various antibiotics, and yet, her condition remains stagnant.

## Discussion

The culprit organism, Clostridium perfringens, is a gram-positive, spore-forming, anaerobic bacilli, which possesses the unique ability to switch gears for means of survival, from aerobic respiration in the presence of oxygen to fermentation in the absence of oxygen [1,3]. It usually colonizes the gut and urinary system as part of the normal flora. Their mode of transmission is through contaminated soil, feces, or food. C. perfringens is known to cause food poisoning, fatal enterotoxaemia, and gas gangrene [3]. However, the clinical picture depends on the immune status and subtype of C. perfringens involved [3]. On the basis of four different exotoxins, the C. perfringens subtypes A, B, C, D, and E have been identified in Table 1.

**Table 1:** Sub-classification of Clostridium perfringens on the basis of exotoxin production

C.Perfringens Subtype	Toxin Produced	Disease
A	Alpha+	Gas gangrene. Enterotoxaemia
B	Epsilon+	Enterotoxaemia in humans and animals
C	Alpha+, Beta+	Fatal enterotoxemia
D	Alpha-, Epsilon+	Enterotoxemia
E	Alpha+, Iota+	Pathogenically unclear

Of the various subtypes listed, subtype A is the most commonly occurring [3]. As per a review of the literature conducted by Hanganu et al., 80% of cases belong to the traumatic subtype [4]. As per the context of our case, traumatic myonecrosis caused due to C. perfringens is deemed to be the most devastating form of clostridial infection due to its rapid, insidious onset [5]. Post-trauma, the initial factor that determines the growth of Clostridium, leading to a vast array of pathological manifestations are the levels of tissue oxygen tension, redox potential, and local pH [1,6]. Tissue equipped with 70 mmHg of oxygen tension or above is known to inhibit growth, whereas any less than 30 mmHg would provide an optimal environment for rapid growth in the contaminated area [1]. Of the 17 toxins produced by C.Perfringens, the α toxin is essential to establish disease [1,6]. The α toxin, with its phospholipase C (PLC) activity, hydrolyzes the lecithin components of cell membrane phospholipids, leading to the eventual destruction of endothelial cell membranes. The subsequent leakage of proteins, fluid, and albumin into the interstitial space results in clinically relevant edema [3,6]. PLC also stimulates conformational changes in the GP IIb/IIIA receptor, thereby accelerating irreversible intravascular aggregates of P-selectin + (activated) platelets, fibrin, and neutrophils [6]. This leads to complete obstruction of local blood flow, resulting in a drop in pH secondary to the anaerobic metabolism adopted by tissues. As a consequence, the redox potential drops to a point suitable for further growth of the bacilli and clinically relevant myonecrosis ensues [4,6]. The rapid spread of infection over connective tissue plains is due to the substantial collagenase activity of the Kappa toxin, allowing rapid spread across fascial planes [1]. Furthermore, other species of the genus Clostridia are also known to cause infections in clinically relevant scenarios. These are discussed in Table 2.

**Table 2:** Clinically relevant associations of other clinically relevant clostridial species

Species	Clinically Relevant Associations
C. sordelli	Fatal shock syndrome, Medication-induced abortion, use of black tar heroin injections, Post-abortive septicemia
C. histolyticum (10%)	Post-traumatic infections, Infective endocarditis, Intramuscular heroin injections, Ulcerative colitis
C. bifermentans (10%)	Septic arthritis, Osteomyelitis, Abdominal infections, Soft tissue infections, Brain abscess, Bacteremia, endocarditis, and empyema

Clostridial myonecrosis is deemed the most devastating type of clostridial infection owing to its ability to rapidly devitalize muscle tissue at a rate of several inches per hour [6]. Given its uncommon incidence in the civilian population, it is important for physicians and surgeons to be familiar with clinical presentation, diagnostic methods, and available treatment modalities. The infection can become well-established in as little as six to eight hours with clinical manifestations presenting one to two days post-trauma [4,6]. The patient may initially experience fever, excruciating pain, local edema, extensive muscle necrosis, hemorrhagic bullae around the wound, fatigue, and dehydration [1-2,5]. Patients present with complaints of severe pain, which is disproportionate to the physical exam findings [5]. At first, there is a lack of superficial inflammation at the site of the injury. As the disease progresses, C. perfringens infects and involves all layers, resulting in marbling and change in color of the skin. On inspection, the appearance of the skin changes from the initial pale to bronze color to purple/red [5]. Furthermore, the decomposition of carbohydrate structural moieties leads to gas formation at the site of the inflicted wound This is responsible for the classical physical finding of crepitations (gaseous gulps) on palpation. Other signs and symptoms include dishwater like wound discharge, foul-smelling musty odor, localized indurations, and eventual sloughing of skin [2]. If left untreated, complications such as septic shock, adult respiratory distress syndrome, disseminated intravascular coagulation (DIC), massive hemolysis, hepatic, and renal failure may ensue [1]. Septic shock and organ failure present in at least 50% of patients; among these, 40% die [6]. The shock in gas gangrene is thought to be mediated due to direct and indirect effects of virulent toxins [6]. PLC induces hypotension via directly suppressing myocardial contractility, leading to a decrement in cardiac output [6]. Furthermore, in a review of 33 cases conducted by Shindo et al., only one case presented with massive intravascular hemolysis [3]. This suggests a prevalence of 3% for hemolysis as a complication [3]. However, the mortality associated with Clostridium-induced hemolysis ranges from 70%-100% [3]. Initial workup in such patients should include complete blood count (CBC), comprehensive metabolic panel (CMP), urinalysis, prothrombin time (PT), activated partial thromboplastin time (aPTT), blood culture, and wound culture [1]. X-ray and ultrasound (US) are useful diagnostic tools to establish the extent of the infection, gas production, and tissue damage [1]. Extensive laboratory diagnostic tests include gram stain smear with germination, protein electrophoresis, and ionogram [1,4]. Hematological investigations may reveal leukocytosis with neutrophilia, anemia with anezoinophilia, and elevated urea levels [4]. Additional tests to screen for the development of sepsis such as arterial blood gases, lactic acid levels, and pre-calcitonin should also be used during clinical monitoring [1].

Clostridial myonecrosis, with its sturdy nature and rapid course of progression, warrants an aggressive therapeutic approach. Therapeutic modalities to tackle the infection include combination antibiotics, surgical consultations with early debridement, IV fluid resuscitation, and hyperbaric oxygen therapy (HBOT) [1]. It is imperative for an early surgical consultation to be obtained and empirical antibiotic therapy to be initiated. Initial surgical measures should include the removal of soil, debris, and washing with hydrogen peroxide and betadine [1,4]. Further surgical measures, such as fasciotomy to relieve compartment pressure, debridement of necrotic tissues, and counter incisions for wound ventilation, are also performed [1,4]. Along with surgical debridement, the early use of empirical antibiotics reduces the fatality rate by 30% [1]. Furthermore, the addition of HBOT further lowers the fatality rate by 5%-10% [1]. Initial empirical therapy includes vancomycin and tazobactam or ceftriaxone with metronidazole [1,4]. However, clindamycin in combination with a second antimicrobial such as penicillin G is strongly recommended due to its ability to halt clostridial exotoxin production [1]. In patients with penicillin allergy alternatives, such as chloramphenicol and cephalosporins, must be used [7-8]. The adjuvant use of HBOT helps improve the bactericidal effect of antibiotics, treats tissue ischemia by causing vasoconstriction to reduce edema, promotes the migration of polymorphonuclear neutrophils (PMNLs) and stems cells to the site of infection [1]. Adverse effects related to HBOT include seizures, Eustachian tube dysfunction, hypoglycemia in diabetics, barotrauma, and gas embolism [1]. Other symptomatic therapies include antipyretics, analgesics, blood transfusions, and the administration of anti-gangrenous serum (400,000-600,000 IU), which is only effective before the fixation of toxins on the tissues [4].

As per the relevant case, it is possible that the fall suffered by the girl might have resulted in minor breaks in her skin. These minor wounds could have acted as entry routes for the inoculation of C. perfringens. However, the early closure of the inflicted extremity at the polyclinic with a cast without obvious radiographic evidence of a fracture could have proved pivotal. The early closure of the wound could have created an optimal anaerobic environment for the growth and spread of C. perfringens. It is also possible that the wound might have been treated with poor aseptic techniques from the outset. Upon the removal of the cast, marked edema along with crepitations and marbling of the skin was noted. These findings were consistent with the clinical presentation discussed above. Our medical team treated the condition aggressively with a combination of extensive surgical debridement and the use of benzylpenicillin along with clindamycin. Upon no improvement in condition, the use of HBOT was initiated. After 40 sessions of HBOT, an improvement was noted. However, due to eustachian tube dysfunction and bleeding from the roots of her hair, the use of HBOT was limited. After a total of 139 sessions of HBOT, the infection has, unfortunately, shown no signs of resolution.

## Conclusions

Traumatic myonecrosis, particularly of clostridial origin, is a pathology rarely encountered in daily medical practice. Given the acute nature of gas gangrene, it is imperative to understand that extensive labs and imaging should not delay the institution of empirical antibiotic therapy and surgical debridement of necrotic tissues. Penicillin G and clindamycin along with early surgical debridement is the need of the hour. It is also important to delay wound closure for all potentially contaminated wounds. As an adjunct, HBOT can further improve the bactericidal activity of antimicrobials and accelerate neovascularization with tissue repair.
